# Effect of Seeding Strategy on the Efficiency of Brand Spreading in Complex Social Networks

**DOI:** 10.3389/fpsyg.2022.879274

**Published:** 2022-05-31

**Authors:** Zheng ShiYong, Li JiaYing, Wang Wei, Wang HaiJian, Umair Akram, Wang Lei, Li BiQing

**Affiliations:** ^1^School of Business, Guilin University of Electronic Technology, Guilin, China; ^2^Management School of Hainan University, Haikou, China; ^3^School of Economics and Management, Wuhan University, Wuhan, China; ^4^School of Management, Jiangsu University, Zhenjiang, China

**Keywords:** seeding strategy, brand community, community structure, complex social networks, brand spreading

## Abstract

In social networks, consumers gather to form brand communities, and the community structure significantly impacts the dissemination of brand information. Which communication strategy is more conducive to information dissemination in different structured brand communities? Considering the above factors, we propose the word-of-mouth (WOM) agent model based on the traditional rumor model and bass model, in which the brand WOM spreading is affected by the user's psychological mechanisms, the network structure, and other factors. Through simulation experiments, the results showed the following: (1) the conclusion of the traditional bass model is no longer applicable to social marketing in brand information diffusion, that is, the effect of external marketing stimulation on information dissemination is limited. (2) The communication effect and the efficiency of information in different structures of the learning-community network are very different. (3) The strategy of hub nodes is not suitable for all types of networks, and the impact of different seeding strategies on the efficiency and effect of brand information dissemination was verified. Finally, the conclusion was verified again using the social network data on Facebook.

## Introduction

Since its emergence, social media has attracted a large number of users to participate in it. The rapid development of big data and streaming media technology has made social media increasingly deep into all aspects of social life, where people communicate and interact, obtain information, or express opinions, and the gathering of users in social media has formed a virtual community (Christakis and Fowler, 2017). According to the 2020 China Social Media Marketing Analysis Report, in the first quarter of 2020, the number of digital users in China was 1.023 billion, and the average number of daily active users was 893 million. The per capita use was 7 h, which was enhanced by 17.8% in 1 year. Many enterprises realize the role of social media in brand spreading, and try to promote the brand through social media marketing, such as using virus marketing strategy and brand information or word of mouth (Bampo et al., 2018). When conducting marketing, such as *via* brand spreading, on social media, enterprises usually use seed strategies based on the consideration of marketing cost and performance. For example, after the enterprise identifies the opinion leaders of user groups, it pushes the brand and product information to these opinion leaders (Libai et al., 2017). Under the incentive mechanism, the opinion leaders have the willingness to spread the brand and share the brand and product information in their social network through word-of-mouth and other communication (Zheng et al., 2022). The advantages of the seed user strategy are mainly reflected in: (1) because of the high brand loyalty of seed users, when they find bugs in products and services, they will actively give feedback to the enterprise and even provide improvement opinions, which is conducive for the transformation and upgrading of the enterprise's products (Wang et al., 2022). (2) Seed users usually have a certain social influence, and the limited marketing cost of enterprises cannot cover all consumers in the whole area, so they can drive the spread of brand word-of-mouth by activating a small number of seed users to achieve better brand communication performance (Montaez et al., 2020). (3) The management of brand communities needs to maintain the activity of users while improving market penetration, and seed users have an active role in preventing user churn and attracting potential consumers (Zheng et al., 2022).

Ludwig et al. (2013) shows that the seed strategy is very effective for enterprise social marketing; therefore (Ludwig et al., 2013), many scholars are concerned about the influence of opinion leaders on word-of-mouth communication effects (such as choosing the hub node as the opinion leader), but there has been little research attention paid to opinion leaders under different network structures and how they can lead to different communication performances, i.e., the impact of the network structure on the social marketing performance (Garber and Goldenberg, 2018). With the rise of more diverse types of social media, such as TikTok, and differences in user relations among different social media platforms (for example, in China, friends on Weibo are more strangers; while friends on WeChat are more acquaintances), leading brand communities that use different types of social media may exhibit different network structure characteristics (Garcia, 2015; Sun et al., 2021). This means that enterprises may have different structures and different natures within the community. For instance, an enterprise with a douban user interest community may also have a fan community, so how to combine these network structures and many kinds of seed strategies to study the influence of social marketing stimuli on different communities is particularly important (Haenlein and Libai, 2017).

In recent years, many studies have focused on the mechanism of information and behavior dissemination using computer simulation technology. For instance, ABMS has been used to study the importance of customer lifetime value and seeding selection for the diffusion of new products/information (Montaez et al., 2020). Garber and Goldenberg (2018) studied the impact of seeding strategy on new product strategy by introducing a proxy model into the mechanisms of increasing the size and the speed of the affected product (Garber and Goldenberg, 2018). Moreover, many studies have been conducted on information and product diffusion based on the bass model and other macro-diffusion models (Garcia, 2015). However, models considering the multidimensional differences among nodes in social impact and social network structure have been rarely discussed (Sun et al., 2021). In other words, the nodes in the default network are homogeneous, thereby leading to a deviation between the research results and the real situation (Haenlein and Libai, 2017).

Given the above deficiencies, this study aimed to (1) describe the results of information diffusion based on different virtual community structures (random network, small-world network, and scale-free network) by ignoring the content of communication; (2) explain how the result of information diffusion is different from the previous research considering the heterogeneity of the network node, the social impact, and the effect of consumer persuasion (Garcia, 2015); (3) match different communication content with different network structures and seeding strategies to optimize the communication effect; and (4) use the ABMS model method to extend the epidemic transmission model and Bass model based on the random network, small-world network, and scale-free network, respectively, employing the social network on Facebook to verify the conclusion.

## Relevant Research

### The Effect of Opinion Leaders

Opinion leaders are a minority who form an important source of information and influence in the team and can influence the majority attitude tendencies (Wang et al., 2022). Although not necessarily a formal group leader, opinion leaders are often well-informed, proficient in current affairs; resourceful, talented in some way; or recognized as having become an opinion leader of the masses or the public (Montaez et al., 2020). In terms of consumer behavior, opinion leaders are people who filter, explain, or provide information to others, who have more knowledge and experience with a certain product or service because of their high level of continued concern (Ludwig et al., 2013). Family members, friends, or well-informed authority figures in the media and the virtual community often act as opinion leaders. Opinion leaders occupy an important role in two-level communication, providing more exposure to a larger crowd and spreading their own re-processed information to others. They intervene in mass communication, accelerating it and expanding its impact (Zheng et al., 2022).

In the study of public opinion leaders, it has been found that different mediums play different roles in the decision-making process, that interpersonal influence is more universal and effective than other types of influence, and can maintain internal opinion and action consistency within a basic group (Garber and Goldenberg, 2018). As a social phenomenon, the opinion leader exists not only in the Western society but also in the process of the neutralization and transmission of different societies, although their appearance may be somewhat different (Garcia, 2015). In the dissemination of information, the information output is not always directed to ordinary recipients, some information only passes to a particular section of recipients, who then pass on the message to the general audience around them (Sun et al., 2021). Even if some messages are passed directly to the general audience to change their attitude and behavior, opinion leaders should explain, evaluate, and guide the message to provide direction or guidance on the situation (Zhu and Zhang, 2010; Ding et al., 2016; Haenlein and Libai, 2017).

### How to Choose Opinion Leaders in Viral Marketing: Seeding Strategy

Viral marketing involves the use of public enthusiasm and interpersonal networks to selectively stimulate users on the network, for instance, by pushing or sending messages to opinion leaders (Jian, 2017; Haijian, 2020). Brand information is spread among consumers through word-of-mouth communication and other behaviors (Na, 2015; Rand, 2018). Previous studies have shown that the success of viral marketing primarily depends on the following four factors: (1) the content of message, the different aspects (interesting, natural, effective, etc.) (Jian, 2017; Schwartz, 2019); (2) the structure of the social network (Yamir Moreno, 2018; Schwartz, 2019); (3) the mechanism of individual information dissemination and the motivation of sharing behavior in the network (Schwartz, 2019); and (4) the strategy of seeding selection (namely the enterprise initially selects which consumer will spread brand information) (Belli and Reyes, 2021). The selection j of the seeding strategy is critical, as the enterprise can achieve better communication with a lower cost using a few seeding users strategically (Tomazic, 2017).

The seeding strategy refers to a small number of consumers (seeding users) who were encouraged to start using the information/product in the early period of the release of information/product (Trusov et al., 2017). Interpersonal communication between consumers facilitates the product or information spread (Stanoevska-slabeva, 2019). Garber and Goldenberg (2018) has emphasized the importance of opinion leaders as seeding users, and researchers have studied many ways of selecting opinion leaders, such as hub nodes or nodes with strong connectivity, or nodes that can bring more profits and benefits to enterprises (Black and Veloutsou, 2017; Garber and Goldenberg, 2018; Tomazic and Udir Misic, 2019). To summarize, the following seeding strategies exist: hubs, or the nodes with the strongest connectivity, and nodes with special location structures (Faraj et al., 2016). For instance, studying those nodes with high proximity to the center, which means less distance from other nodes, makes it possible to better observe the flow of information. In physics, the node that describes the importance of the node also has the node with a high intermediary center, which means that the node is important to the information flow. The aggregation coefficient describes the level of interaction between a node neighbor node (Gittell et al., 2008). Moreover, more recent research has been conducted by Dodds et al., who proposed a node with a high k-kernel coefficient, that is, the node at the core of the network structure (Dodds, 2017). In this study, we synthesize these indices to evaluate the influence range and efficiency of each index on the information transmission in different network structures.

In recent years, many scholars have begun to study the diffusion of information and products by simulation. The existing communication models fall into two categories. The first category is the ordinary differential equation similar to epidemic spread (SIR and SIS) and rumor propagation (Yu and Hageman Blair, 2016; Zhang et al., 2016; Akram et al., 2017). This kind of model is widely used in the field of epidemic and complex network dynamic systems. The other is the generalization of the macroscopic diffusion model, which is dominated by the bass model (Garcia, 2016). Table 1 summarizes the research related to epidemic, information, and product diffusion in recent years. These two models have a similar premise to the social marketing research: (1) ignore the heterogeneity of network nodes, that is, every consumer in the network has the same probability of changing the state, completely independent of network structure, and other factors (Duan et al., 2015; Kawamoto and Rosvall, 2015; Akram et al., 2018); (2) the research object is based on the individual, for instance, hub nodes, opinion leaders, etc. are more concerned with the effect of node-local network characteristics on diffusion behavior, and do not consider the impact of community network structure on the communication effect (Yang et al., 2014; Okamoto, 2016; Black and Veloutsou, 2017). In this study, based on the structural characteristics of the consumers in the network, the persuasion information theory and the social impact theory, we improve the homogeneity of system nodes in previous models (Tomazic and Udir Misic, 2019). To do this, we consider the probability that each consumer in the network is affected by word-of-mouth information because of their difference in position and the probability of external marketing stimulation persuasion (Berger, 2014; Goel, 2018; Timothy, 2018; Akram et al., 2020).

**Table 1:** Research on the impact of product or information dissemination based on the complex network.

**Related studies**	**Keywords**	**Data source**	**Network structures**	**Criteria for seeding users**	**Research results**	**Criteria for subject investigate**
Chakravorti (2014)	Networks; technology policy diffusion; strategy	Facebook	Small-world networks	–	Propagation ranges	Individuals
Dodds (2017)	WOM; Opinion Leadership; Diffusion Innovation	Laboratory experiment	Stochastic networks	Hub	Outbreak range	Individuals
Garcia (2016)	Community Detection; BoCluSt;Computer simulation	NCBI database	Reality network	Opinion Leader: hub	Usage	Individuals
Guidi et al. (2015)	Distributed Online; Social Networks; Data availability	Facebook'14 data set	Experiment: without consideration of structure	Interested user	Propagation ranges	Individuals
He et al. (2014)	Community Networks; Models; Theoretical	Palla's website	Reality network	Hub/Intermediate Center/K-nuclear coefficient	Propagation ranges	Individuals
Kheirk et al. (2016)	Community detection;bipartite networks;Markov times	Flows on a citation network.	Small-world networks (undirected)	Hub/Intermediate Center	Diffusion range	Individuals
Bampo et al. (2018)	Viral marketing, information diffusion, social networks	Artificially generated networks	Reality network	Hub/Intermediate Center	Application amount	Individuals
Okamoto (2016)	Complex network; Community; Local detection; Short-term memory	Artificially generated networks	Small-world networks (undirected)	Hub	Application amount	Individuals
Ourresearch	Seeding strategy; brand community; community structure; complex social networks	Simulation experiment;Facebook	Random networks/small-world networks/scale-free network	Hub/proximity/ aggregation coefficient/K-nuclear coefficient	Diffusion range;time cost;communication efficiency	Groups

*To be arranged by the author*.

### Statistical Features of Virtual Community Network Structure

Virtual communities are also known as online communities. From the user's perspective, virtual communities are divided by sociological domains into the following types according to their function and nature: (1) relationship communities, (2) transactional communities, (3) interest communities, and (4) fantasy communities (Okamoto, 2016; Yang et al., 2016a,b; Akram et al., 2021). Based on the functional division of the community, some studies have classified it into a discussion community, task-based community, mixed community, etc (Beckett, 2016; Tomazic and Udir Misic, 2019). Our research primarily studies the information diffusion mechanism in the first three kinds of communities (relational community, transactional community, interest community) based on the user's perspective. In addition, in the field of complex network science, there are several related studies on the statistical structure characteristics of virtual communities. Zhang and Newman (2015) analyzed the statistical characteristics of user friendships on the Sina blog and the school net, and also verified the universality of small-world and scale-free characteristics and the heterogeneity of virtual community networks (Zhang and Newman, 2015). The research results of Faraj et al. (2016) show that the nodes of online social networks are distributed by the power rate, while Okamoto (2016) suggest that the degree of nodes in online social networks is composed of power distribution and exponential distribution (Faraj et al., 2016; Okamoto, 2016). Accordingly, there is no consistent conclusion on the virtual community network structure. Therefore, this study chooses three kinds of network structures as the representatives of the community structure to study social marketing strategy. These three networks are random networks, small-world networks, and scale-free networks (Xiao and Huang, 2015). In small-world networks, most of the nodes are not adjacent to one another, but most can be accessible in a few steps from any other nodes. In scale-free networks, most “normal” nodes have very few connections, while a few “hot” nodes have a large number of connections. In random networks, the nodes, arrows, and traffic all have a degree of uncertainty, and the activities composing the network graph can also be random. Furthermore, network data from Facebook were selected to verify these networks. The information on the network data collected from Facebook is as follows: nodes, 4,039; edges, 176,464; closeness centrality, 0.2761; average degree, 43.69; betweenness centrality, 0.00067; clustering coefficient, 0.6055.

By constructing the ABMs model of information diffusion (Xia et al., 2015), which is closer to the real situation, and considering the impact of the selection of multiple seeding strategies on the time, scope, and efficiency of information transmission under different network structures, this study provides theoretical guidance close to reality based on the scope and efficiency of viral marketing strategy (Yoldemir et al., 2016).

## Materials and Methods

One of the obvious advantages of using the proxy model is that it is possible to observe the emerging macro results of groups based solely on the interaction mechanism between individual users without knowing the macro dynamic mechanism (Yamir Moreno, 2018). Moreover, in many situations of market research, users have complex interactions, which are difficult to describe using a traditional mathematical model (Stanoevska-slabeva, 2019). Thus, an increasing number of studies in the field of marketing and communication have begun to use the agent model to study the diffusion of information. In addition, Garber and Goldenberg (2018) provided strict guidance and specifications for using the agent model in information diffusion research (Garber et al., 2019). Next, the selection of the network structure and the building of the model are introduced (Schulke and Ricci Tersenghi, 2015; Zhang and Siddhartha, 2016).

### Selection of Network Structure

Previous studies have shown that online communities are scale-free and small-world (Tomazic, 2017; Garber and Goldenberg, 2018). Accordingly, to study the impact of different network structures on the diffusion of information, under the premise of controlling network size and network density, the network structure of the random network (ER), small-world network (WS), scale-free network (SA), and real online social network Facebook (snap.stanford.com) were selected. Table 2 shows the structure information of this research network.

**Table 2:** Characteristics of network structure.

	**Networks**	**Size**	**Average degree**	**Closeness centrality**	**Betweenness centrality**	**Clustering coefficient**
1	ER	1,000	9.814	0.306	0.00228	0.0090
2	WS	1,000	10	0.2976	0.00237	0.0959
3	SA	1,000	9.95	0.3373	0.00199	0.0394
4	Facebook	4,039	43.69	0.2761	0.00067	0.6055

*All networks in the table are undirected networks*.

### Model Destruction

This study focuses on the dynamic evolution of discrete-time systems. The proposed model assumes that there are only three kinds of nodes in each stage: (spreader) value: 1, (ignorance) value: 0, and (silence) value: −1. Spreader refers to users who know the brand information and are willing to spread the information in the network, ignorance refers to the users in the network who do not know the information; and silence refers to the users who know the information but do not want to spread it again. In each phase, users interact only with their associated learners (van der Lans et al., 2018).

**Figure 1:**
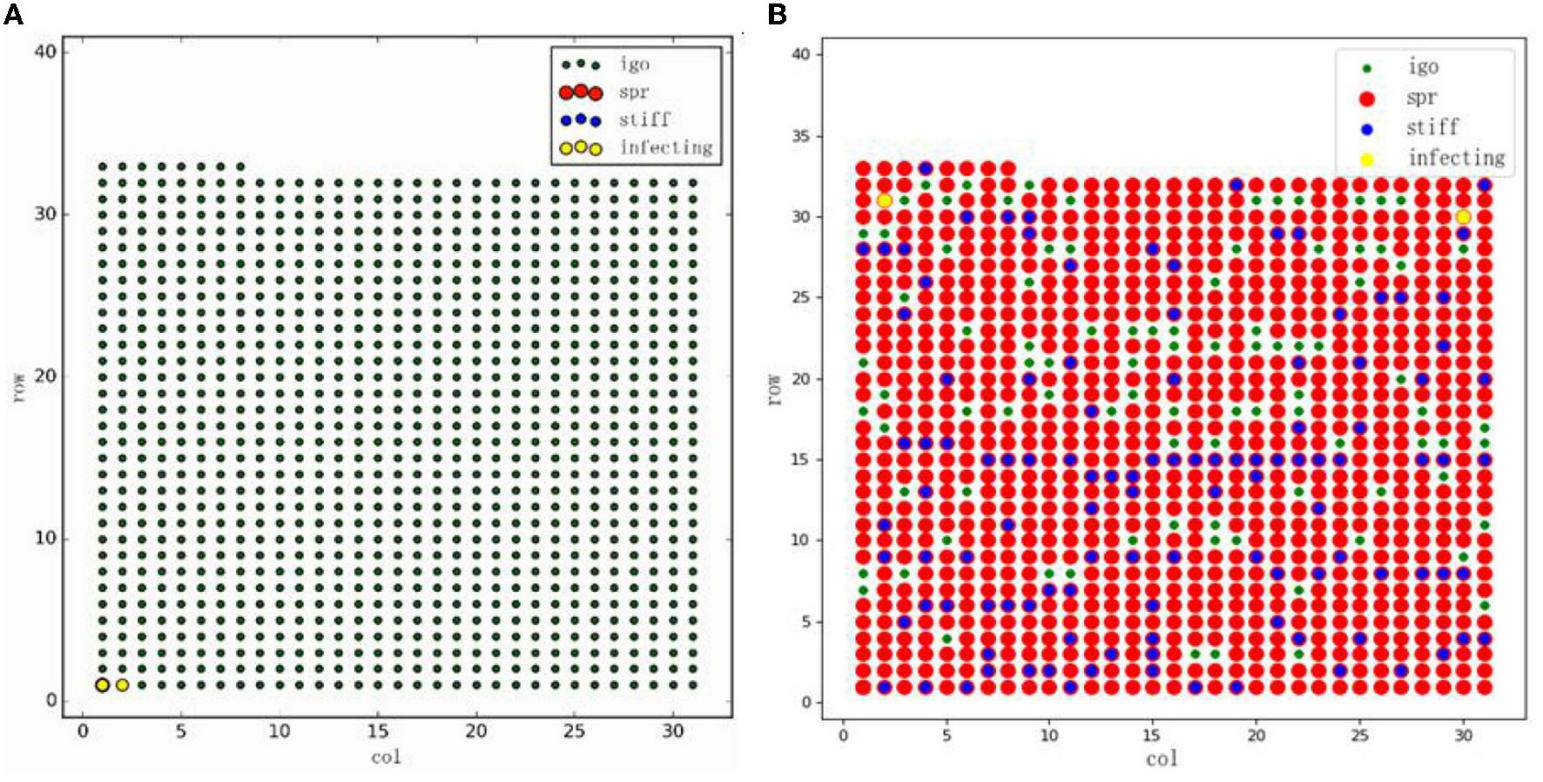
Diffusion simulation process.

In each stage, there are only two types of state changes: (1) spreader changes to silence, and (2) ignorance changes to a spreader. The communication process terminates when there is no longer a spreader in the system. The initial state and the mid-diffusion period are shown in Figures 1A,B, respectively. The red node suggests that the seeding user (spreader) value is 1, the green node means that the susceptible user (ignorance) value is 0, the blue node indicates that the silent user value is −1, and the yellow node indicates that the spreader is spreading.

Based on the bass model, the transition from the ignorance to the spreader is affected by two factors (Haenlein and Libai, 2017; Jian, 2017; Trusov et al., 2017):

(1) The external information stimulation δ. Based on the bass model, this study considers the consumer persuasion theory. Since the hub node has more information, it has a stronger judgment ability and information screening ability. Thus, nodes with high degrees of centrality are more likely to use persuasive information, so it is assumed that the same external stimulus has less influence on nodes with a high degree of centrality. Selecting


$$\begin{array}{l} \left(1-\frac{k}{\max\left(k\right)+\xi }\right)\;\epsilon \;\left(0,1\right) \end{array}$$

as the attenuation coefficient of the information stimulation to different nodes, ξ(>0) → 0, the impact of external information stimulus on ignorance nodes is inversely proportional to its degree of centrality, namely


$$\begin{array}{l} \left(1-\frac{k}{\max\left(k\right)+\xi }\right)\delta \end{array}$$

and max(k) is the maximum degree of nodes in the network.

(2) The internal influence is *q*_*i*_, i.e., the probability that users will be affected over a period of time by interacting with the spreader. Given the herd behavior of social impact, it is assumed that the ignorance is in contact with the spreader. The probability of transforming into a propagator refers to the proportion of the propagator node in its neighbor node, which is recorded as *q*_*i*_.

We aimed to study the impact of different seeding selections on the effect of network marketing with different structures. At the beginning of the spread, the number of nodes is N, and only one spreader is present. This means the number of ignorance is N-1. Then, the communication process is as follows:

(1) At the time t, the proportion of spreader, ignorance, and silence are *s*(*t*), *i*(*t*), and s(t). Thus, *s*(*t*)+*i*(*t*)+*r*(*t*) = 1,$s\left(0\right)=\frac{1}{N},\;i\left(0\right)=\left(1-N\right)/N.$

(2) At each stage, the spreader interacts with all neighbor nodes. The probability λ_*i*_ that the ignorance i receives information and becomes a spreader is associated with two factors: (i) external factors, such as external stimulus intensity δϵ(0, 1], namely the probability that an ignorant person will be transformed into a spreader because of the information stimulus of the enterprise; (ii) given the conformity of individual, the probability of ignorance herd behavior is considered proportional to the spreader in the neighbor node, so the parameter *q*_*i*_ might be set as the proportion of the spreader in the neighbor node. In other words, the probability of the ignorance occurrence of herd behavior is proportional to that of the spreader in the neighbor node.


(1) $$\begin{array}{r} q_{i}=\frac{K_{is}}{K_{i}} \end{array}$$

where *q*_*i*_ is the following parameter of node i, *K*_*is*_ is the propagator in the neighbor node of node i, and *K*_*i*_ is the center of the degree of node i.

According to the bass model, the probability λ_*i*_ of ignorance interaction with the spreader to become a spreader is 1 minus the probability that all external and internal factors have no effect on it, that is:


(2) $$\begin{array}{r} \lambda_{i}=1-\left(1-\;\delta_{i}\right){\left(1-q_{i}\;\right)}^{K_{is}}=\\ 1-\left(1-\left(1-\frac{k}{\max\left(k\right)+\xi }\right)\delta \right){\left(1-\frac{K_{is}}{K_{i}}\right)}^{K_{is}} \end{array}$$

(3) The spreader changes to silence after interacting with the spreader or the silent person with probability α (α can be understood as the sensitivity coefficient of the spreader to information).

(4) When there is no longer a spreader in the network, the communication process terminates.

The results of the brand information transmission primarily consider the following three aspects: (1) the scope of information transmission, that is, the proportion of silence in the network node when the communication is terminated; (2) the time cost of information transmission, that is, the number of iterations at the end of the communication; (3) the efficiency of communication, that is, the average E for each stage, as defined in Table 3.

**Table 3:** Information dissemination effect.

**Results variables**	**Meaning**
Scope of transmission S (scope)	Proportion of silence at the end of transmission
Transmission time T (time)	The time to spread brand information throughout the network
Transmission efficiency E (efficiency)	At the end of spread, S/T can represent the average range of infection per transmission.

*All networks in the table are undirected networks*.

## Results

For the simulation, a seeding input model was selected for the simulation analysis each time (Lehmann and Esteban-Bravo, 2016). The sensitivity of parameters α and δ to the system was first analyzed. Then, we discussed the effects of seeding selection on propagation results in different network structures based on the sensitivity test results and the fixed parameters α and δ. Python is a typical object-oriented, interpretive program computing language and it is effective to simulate ABMS. Thus, Python was used to execute the whole simulation process.

### Parametric Sensitivity Test: Effects of Marketing Stimuli and Spreader Immune Coefficient on the System

**Figure 2:**
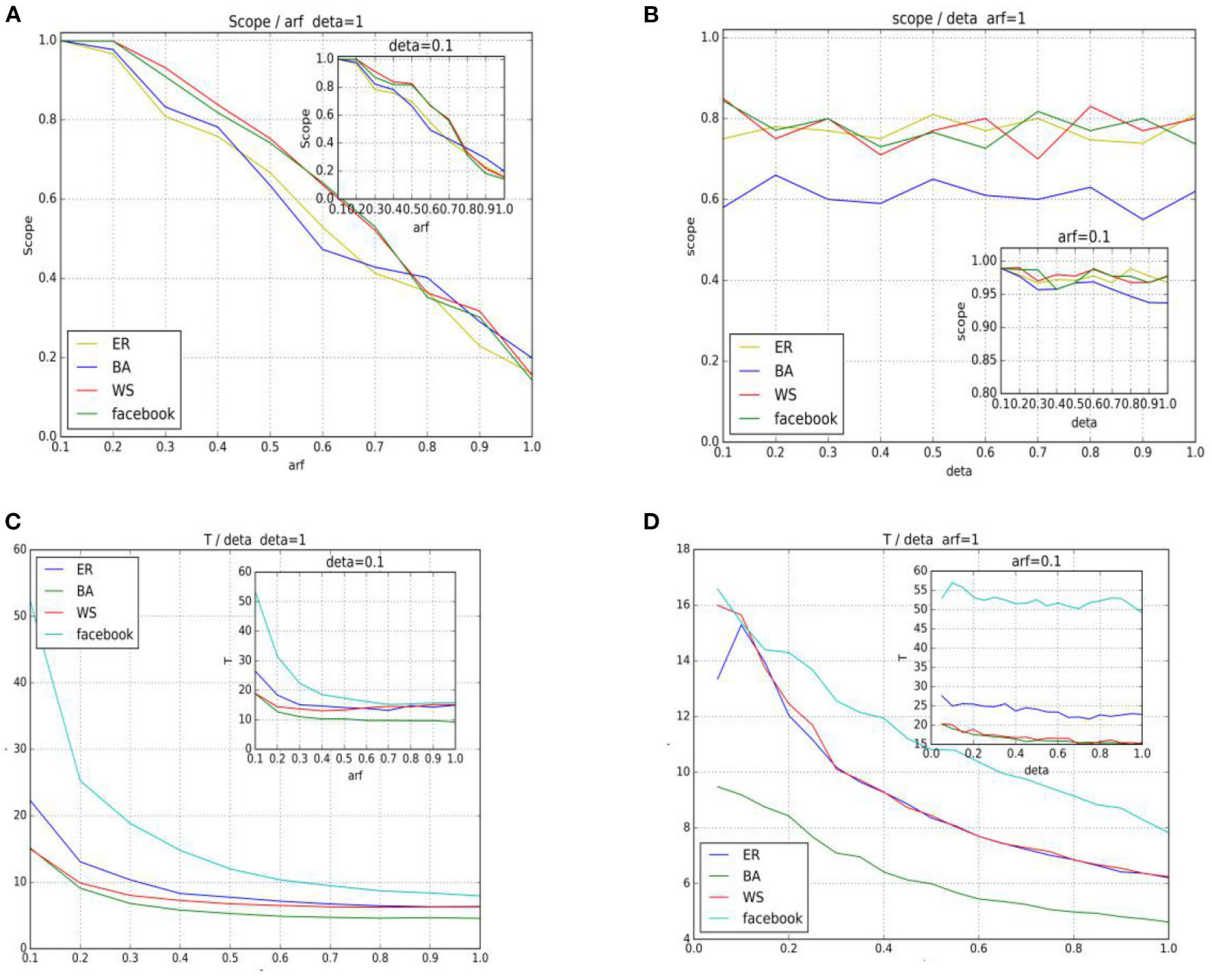
Simulation results. **(A,C)** Represents the fixed δ, the effect of the α change on the information diffusion range, and the time required for the whole diffusion process. **(B,D)** Is the fixed α, the time of the variation of the information diffusion range, and the whole diffusion process.

The following is a study of the effects of brand information stimulus δ and spreader immune coefficient α on the spread range S and propagation time T, where 100 seeds were randomly selected in three different types of networks. One seeding node was selected for each propagation experiment, and each seeding node was repeated 20 times. After the input from different parameter groups (Table 4), the average transmission range and efficiency were calculated, and Figure 2. was obtained. For instance, when δ was fixed in Figure 2A, the propagation range decreased with the increase in α in all three networks. In general, the small-world network had a wider range than the scale-free network. As shown in Figure 2B, the scale-free network had the shortest time (the fastest) among the three types of networks, and with the increase in α, the propagation time of the three networks was close to stable.

**Table 4:** Parameter settings.

**Parameters**	**Range**
δ	0.1,0.2,0.3,0.4,0.5,0.6,0.7,0.8,0.9,1
α	0.1,0.2,0.3,0.4,0.5,0.6,0.7,0.8,0.9,1
Seeding type	Degree centrality, intermediary centrality, proximity centrality, aggregation coefficient, k-kernel coefficient
Network type	ER, SA, WS, Facebook

As shown in Figure 2C, the marketing stimulus δ has a limited effect on the range of communication to some extent when α is fixed (except for α = 1), but it will affect the time of dissemination of information, as shown in Figure 2D. Accordingly, when enterprises focus on the scope of marketing information, the importance of interpersonal communication should be noted (e.g., word-of-mouth communication).

### Impact of Different Seeding Strategies on the Effect and Scope of Information Diffusion

Next, to study the impact of seeding selection on the propagation effect in different network structures (Na, 2015), 100 seeds were randomly selected from each of the 3 networks, and one seeding, i1, was selected to propagate in each experiment. The propagation range S and time T at the end of propagation were obtained, and the experiment was repeated 20 times on the same seed. The average propagation range and time of each seeding were yielded by *SE*_∞_(*i*1). The propagation effect vector of each seeding can be obtained by repeating 20 experiments on each seed, namely 〈*SE*_∞_(*i*1), *SE*_∞_(*i*2), …*SE*_∞_(*i*100)〉 (average obtained after 20 operations), to examine the value of each seeding attribute X (i) and propagation results of each node, see: the Pearson correlation coefficient of 〈*SE*_∞_(*i*1), *SE*_∞_(*i*2), …*SE*_∞_(*i*100)〉ρ(*SE*_∞_(*i*), *X*(*i*), where X (i) denotes a five-dimensional vector:

X(i) = 〈*Degree*_*i*_, *Betweenness C*_*i*_, *Closeness C*_*i*_, *Kernal*_*i*_, *Clustering*_*i*_〉. In random networks, small-world networks, and scale-free networks, correlation coefficient matrices were developed, as shown in Table 5.

**Table 5:** Correlation coefficient table.

**WS**	**Degree**	**Clustering**	**Closeness**	**Betweenness**	**Core**	**S**	**E**
Degree	1	−0.38	0.93	0.92	0.62	0.57	0.06
Clustering	−0.38	1	−0.5	−0.46	−0.22	−0.1	−0.15
Closeness	0.93	−0.5	1	0.88	0.67	0.57	0.05
Betweenness	0.92	−0.46	0.88	1	0.41	0.45	0.05
Core	0.62	−0.22	0.67	0.41	1	0.47	−0.03
S	0.57	−0.1	0.57	0.45	0.47	1	−0.53
E	−0.06	0.15	−0.05	−0.05	0.03	−0.53	1
ER network	Degree	Clustering	Closeness	Betweenness	Core	S	E
Degree	1	0.12	0.92	0.96	0.75	0.56	−0.16
Clustering	0.12	1	0.1	0.04	0.22	0.14	−0.16
Closeness	0.92	0.1	1	0.88	0.81	0.56	−0.16
Betweenness	0.96	0.04	0.88	1	0.63	0.5	−0.1
Core	0.75	0.22	0.81	0.63	1	0.44	−0.16
S	0.56	0.14	0.56	0.5	0.44	1	−0.77
E	0.16	0.16	0.16	0.1	0.16	−0.77	1
SA network	Degree	Clustering	Closeness	Betweenness	Core	S	E
Degree	1	−0.3	0.87	0.94	0.6	0.6	−0.19
Clustering	−0.3	1	−0.46	−0.44	−0.29	−0.04	−0.04
Closeness	0.87	−0.46	1	0.9	0.6	0.56	−0.23
Betweenness	0.94	−0.44	0.9	1	0.47	0.51	−0.15
Core	0.6	−0.29	0.6	0.47	1	0.46	−0.13
S	0.6	−0.04	0.56	0.51	0.46	1	−0.69
E	0.19	0.04	0.23	0.15	0.13	−0.69	1

*S means the range of information diffusion. E refers to the efficiency of information diffusion, as defined in Table 2. Core is the number of k kernel layers, and the coefficient is the value after standardization (Red font means statistically significant)*.

As shown in Table 5, the seeding aggregation coefficients in the three different networks are not significant for the spread of word-of-mouth information and several other types of central indicators. Overall, the degree, proximity to centrality, intermediary centrality, and the number of audits are associated with the spread of information. It is also suggested that in the small-world network and scale-free network, the scope of information dissemination and dissemination efficiency are negatively correlated. In other words, the scope of enterprise brand information dissemination comes at the cost of communication efficiency.

In many cases, enterprises should consider the scope and efficiency of information dissemination, which depends on the enterprises' reliance on the scope of information dissemination and the efficiency return when they conduct a socialized marketing strategy. Here, we standardize the propagation range S and the efficiency E and obtain 〈*S*〉,〈*E*〉. Then, we let the enterprise distribute the weight between them to be η and (1- η), where η ∈ [0, 1], assuming the cost corresponding to the external marketing stimulus is c ^*^ δ, where c is a non-negative constant. It can be assumed that the enterprise faces a profit function in each socialized marketing strategy:


$$\begin{array}{l} \pi =\eta \langle S\rangle +\left(1-\eta \right)\langle E\rangle -c\delta ,\eta \in \left[0,1\right] \end{array}$$

For instance, when enterprises think that the range of transmission is as important as the efficiency of transmission, the value η = 0.5 is taken. Then, to study how to select the right seeds for transmission, π = 〈*S*〉+〈*E*〉 is calculated to reach its largest value. At this time, we use π to make a regression analysis of seeding indexes. The results are shown in Table 6.

**Table 6:** Regression of Π for the values of various seeding attributes.

**DV= **π**(**η =** 0.5)**	**ER**		**SA**		**WS**	
**Indexes**	**Coefficients**	**Pr(>|t|)**	**Coefficients**	**Pr(>|t|)**	**Coefficients**	**Pr(>|t)**
Intercept	−0.43604	0.3338	0.249035	0.29678	−0.23524	0.4315
Degree	0.014627	0.1586	0.010196	6.6e-05^***^	0.016627	0.1387
Clustering	0.207787	0.0578	−0.04741	0.51501	1.407259	0.6524
Closeness	4.155813	0.0259^*^	0.486681	0.16363	1.125012	0.0259^*^
Betweenness	−0.26904	0.1219	−1.26226	0.00103^**^	−0.31050	0.04219
Core	−0.00167	0.9201	–	-	0.0219	0.0239^*^

*The dependent variable is the weighted average of the range of propagation and the propagation efficiency*.

* *Denotes p < 0.05*;

** *denotes p < 0.01*,

*** *denotes p < 0.00*.

It is suggested that when the enterprises think that the transmission efficiency and spread range are equally important, the hub node remains important in the scale-free network. However, in the small-world network, the hub node is no longer the best seeding node, and the center of the node is more valuable for the overall effect of communication. Considering that the real online social network is both small-world and scale-free, this study uses Facebook social network data as an example to study the effect of seeding strategy on the spread range and efficiency.

According to the definition of a small-world network, Table 7 suggests that the average aggregation coefficient of Facebook is 0.6055 and the length of the characteristic path is 0.2761 with obvious small-world properties. When only considering the scope or equilibrium scope and efficiency, the degree of centrality does not significantly impact the communication effect, proximity is the better seeding index, and the intermediary centrality can inhibit the transmission effect. When only considering the propagation benefit, the hub node contributes to the propagation, and the proximity to the center significantly impacts the comprehensive effect of communication. Moreover, since Facebook has more obvious small-world properties, the k-kernel number can also positively impact the propagation efficiency.

**Table 7:** Regression of Facebook network transmission effect to seeding attributes.

**DV=**π****	**DV=** **π(η =** **1)/DV=S**		**DV=π(η =** **0.5)/DV=S+E**		**DV=π(η =** **0)/DV=E**	
**Indexes**	**Coefficients**	**Pr(>|t|)**	**Coefficients**	**Pr(>|t|)**	**Coefficients**	**Pr(>|t|)**
Intercept	−0.67664	2.68e-10^***^	−0.71074	1.6e-10^***^	−3.41E-02	3.50e-14^***^
Degree	0.00055	0.1312	0.000579	0.12523	2.89E-05	0.05409
Clustering	0.031686	0.631	0.032072	0.6389	3.86E-04	0.88655
Closeness	4.615281	<2e-16^***^	4.823653	<2e-16^***^	2.08E-01	<2e-16^***^
Betweenness	−4.14439	0.0037^**^	−4.33699	0.00337^**^	−1.93E-01	0.00104^**^
Core	0.000828	0.3432	0.000994	0.27274	1.65E-04	7.11e-06^***^

The first row in the table is a dependent variable. *Denotes p < 0.05;

** *denotes p < 0.01*;

*** *denotes p < 0.001. Coefficient is the standardized value*.

From this perspective, whether hub nodes or other central indicators of word-of-mouth transmission are the best, they must be combined with the network structure and the focus of the enterprises. For the other values of η, the same method can be applied to select the appropriate seeds.

## Discussion

Based on the mentioned results and discussion, with the development of the Internet, the high interactivity of enterprise users forms different brand communities with different structures. Thus, it is difficult for enterprises to capture the fragmentation of behavior data. On that basis, this study can help enterprises simplify their information diffusion strategy and conduct marketing within the community formed by users. Based on the results, enterprises should consider factors, such as social learning among users (application of persuasive information), conformity, and impact of network structure on the communication mechanism in the process of information dissemination. These factors have major implications in theoretically guiding enterprises to empower users of the brand community with marketing.

By building an online community information diffusion agent model, this study analyzed the selection of different seeding strategies under different structure networks and set the heterogeneous parameters of agents and their information attributes. In other words, considering the node herd behavior, persuasion effect, and other factors, to modify the user interaction behavior, the multiagent simulation model was built under the framework of the random network, scale-free network, and small-world network, and the simulation analysis was conducted. This study analyzed the impact of different seeding strategies on the scope and efficiency of online community socialization marketing. Combined with the abovementioned results, the main conclusions of this study are as follows:

(1) Previous search on the socialized marketing seeding strategy has only considered the new product or the brand information diffusion of the existing embedded network of the enterprise (Wang et al., 2022). The effect of the network structure on the propagation effect was ignored (Faraj et al., 2016; Garcia, 2016; Libai et al., 2017; Haijian, 2020; Montaez et al., 2020; Wang et al., 2022; Zheng et al., 2022). This study considers the effect of different network structures, such as random networks, scale-free networks, and small-world networks, as well as the effects of the three typical different network structures on the seeding strategy selection. It was found that after controlling the network density and network size, the diffusion range of information in the small-world networks was wider than that in the scale-free networks. In terms of the diffusion time, the information spreading was faster in the scale-free networks than in the small-world networks.

(2) Based on the sensitivity test of marketing stimulus δ and communicator to information value perception parameter α, external effects (e.g., enterprise marketing stimulation) limited the impact on the scope of marketing information dissemination. The diffusion effect of information was primarily caused by the interpersonal influence inside the network. In the case of a fixed marketing stimulus, the scope of information diffusion decreased with the increase of the communicator's perception of the value of information. These findings suggest that the higher the probability that the communicator becomes silent, the lower the level of acceptance of information, the smaller the spread, and the more time it will take.

Based on the social network theory, the greater the degree of a node, the greater its influence. Therefore, in current studies of seed strategies, large users are usually selected as seeds, such as hub nodes. Related studies are summarized in Table 1. Without considering the content of propagation, this study considers the impact of the selection of five kinds of seeds on the efficiency and effect of information diffusion in networks with different structures, which is an extension and supplement to the previous seeding strategies.

First, this study considers an index based on local statistical properties: (1) the most connected indicator, including the degree of centrality and the hubs that have traditionally been the most studied, and (2) the index of clustering degree, including the aggregation coefficient.

Second, the index is based on global network characteristics: (1) the local importance index, including the intermediary centrality. This index means that the node controls the information transmission between the nonadjacent participants. The higher the value, the better the control of information flow. (2) The proximity to the central indicator, indicating how difficult it is for a node to interact with all other nodes. (3) The k-kernel layer coefficient, which includes both the quantitative and qualitative information of the neighbor nodes.

Based on the above seeding strategy, this study also considered the scope of the virus marketing strategy and the tradeoff of the transmission efficiency, which can theoretically highlight the cooperation between the expected target, the seeding strategy, and the existing network structure of the enterprise. For instance, in SA networks, traditional research conclusions are still used. In particular, the choice of opinion leader hub node contributes the most to communication. However, for the WS network, when only considering the scope of communication, the hub node is not the most ideal seeding node. The closer the central index, the higher the level of interaction between the node and the neighbor node will be, and the more effective the propagation range will be. However, the k-kernel coefficient is also vital when synthetically considering the effect and efficiency of propagation. This suggests that the quality of the seeding user's “friend” is also the information that the seeding user should consider. Finally, this study selected the data from Facebook, the world's most popular data platform, and verified the corresponding seeding selection strategy in the typical social community network's social marketing strategy.

## Research Contributions

### Theoretical Contributions

(1) This study expands the research related to seed user selection strategies in online marketing. Previous studies that only considered the proliferation of new products or information embedded in a company's existing network (Haenlein and Libai, 2017), mainly based on user influence as the basis for seed user selection, usually treat social media as social networks with the same structural characteristics, ignoring the heterogeneity of network nodes, i.e., assuming that each consumer in the network has the same probability of state change and is completely unaffected by network structural factors; however, the network structure of social media is changing due to the differences in user relationships and network evolution stages. Therefore, in the process of brand communication, the seeding strategy should be selected based on the network structure.

(2) This study deepens the research related to brand communication as well as word-of-mouth extension. Previous studies on word-of-mouth communication have been based on individuals (Faraj et al., 2016), for example, hub nodes, opinion leaders, etc., and have more often considered the role of individual consumers' local network characteristics on word-of-mouth communication behavior, ignoring the impact of the overall social network structure on the effectiveness of word-of-mouth communication. In this study, based on the structural characteristics of consumers in the overall social network, we use persuasion knowledge theory and social influence theory to improve on the homogeneity of system nodes in previous model studies, i.e., (Garcia, 2016), we consider that each consumer in the network has different probabilities of being influenced by word-of-mouth messages and of being persuaded by external marketing stimuli due to their different locations.

(3) This study explores the application of social influence theory and value perception theory in the field of brand communication. This study finds that users' states are influenced by the states of neighboring nodes, which is consistent with the findings of previous studies (Wang et al., 2022), and users' willingness to participate in brand communication (word-of-mouth communication) is also influenced by the value of information. After the information redundancy is high and all surrounding users already have the same brand information, consumers' perceived value of the brand information decreases, and consumers' willingness to forward it weakens. Therefore, it is important to pay attention to the role of consumer groups and the attractiveness of brand information to consumers.

### Management Significance

With the development of the mobile Internet, brand communication in social media has received attention from many companies. This study on brand communication in social networks helps companies correctly recognize the role of seed users and selection strategies in different scenarios and provides references for marketing activities, such as brand communication.

(1) It helps companies correctly recognize the role of seed users. This study finds that the selection of seed users has a great impact on the efficiency of brand communication. However, a seed user is not effective in all network structures. That is, the performance of a seed user depends largely on the network environment he is in. Therefore, companies need to fully consider the influence of the network environment when implementing seeding strategies. For example, when a company creates a brand community, the brand community at the initial stage of creation is a random network, and as users continue to join, the community network becomes a small-world network. When the community grows to maturity, the community network becomes a scale-free network. Then at different stages, to get better brand communication performance, companies should go for different seed selection strategies.

(2) It helps companies manage the brand communication performance. When we do not consider the marketing cost and only pursue the brand communication range, we can choose the most influential users as seed nodes to achieve the brand information coverage in the whole area through a longer communication time. However, the more influential seed users means the brand managers need to pay higher marketing costs, for example, when hiring celebrity endorsement, the more influential stars, the higher the cost; however, not all influential stars means a better brand communication effect. Therefore, for enterprises, if we are pursuing the cost performance of brand communication, we can consider the influence of individual seed users, marketing costs, and the efficiency of brand communication.

(3) It helps companies plan effective brand content. This study finds that consumers' perception of the value of brand content affects their willingness to spread it. If friends around them have already acquired the brand information, it will reduce consumers' willingness to spread the brand. This means that companies should frequently construct novel brand advertisements and update brand messages to keep them attractive to consumers as a way to increase their willingness to communicate.

## Conclusion

This study used the ABMS model to study the effect of seeding strategy selection on the effectiveness and efficiency of social marketing strategy in virtual communities. This study has the following limitations and shortcomings. First, by selecting the group concept “online community” as the research object and considering the impacts of different network structures on the behavior of information diffusion, we studied the random network, SA scale-free network, and WS small-world network under the controlled network density. However, some studies have pointed out that the aggregation of users in social networks can form communities, community internal nodes are closely related, nodes between communities are sparse, and the division of communities also affects the dissemination of information messages (Zheng et al., 2019). Combined with the conclusions of this study, the propagation efficiency of the selected seed nodes is located in the same and different communities. Therefore, in future studies, we can analyze the transmission efficiency under different community division rules. Second, this study considered the heterogeneity of nodes in the network structure but did not explore the difference in the results in different situations from the psychological mechanism of consumer behavior. Third, we studied the diffusion mechanism of information in the virtual community. A deviation remains between the model setting and the real communication mechanism, which can be further improved according to the psychological mechanism of consumer behavior. Fourth, the data source of this study is a simulation experiment, which is a relatively singular source. At present, research in the field of consumer behavior mostly adopts questionnaires and secondary data (Zheng and SuPing, 2019; Zhu et al., 2019). Therefore, if the results of this experiment could be verified through primary or secondary data in the future, the research conclusions may be strengthened. Finally, when the community is studied as an object, different types of community relations can also be considered, including the transaction community, interest community, and interactive community. All of these topics should be further studied.

## Data Availability Statement

The original contributions presented in the study are included in the article/supplementary material, further inquiries can be directed to the corresponding author/s.

## Author Contributions

ZS conceived, designed the study, and wrote the manuscript. LJ and WL collected the data. UA analyzed and interpreted the data. WH provided funding support. LB and WW contributed to the revise of the manuscript. All authors contributed to the article and approved the submitted version.

## Funding

This research was funded by the following funds: Guangxi Science and Technology Base and Talent Special Project: Research on the incentive mechanism of user information sharing in live e-commerce-based on social capital perspective (No. 2020AC19034), 2021 Guangxi Education Planning Project: Research on the Influence of Learning Communities in Information Technology Environment on Users' Online Learning Behavior (No. 2021A033); Research on the Influence of Short Video Sharing on Chinese Cultural Identity of International Students in China-Taking Jitterbug as an Example (No. 2021ZJY1607), 2022 Guangxi Degree and Postgraduate Education Reform Project: Research on Cultivating Innovation and Practical Ability of Postgraduates in Local Universities in Guangxi (No. JGY2022122), Teaching reform project of Guilin University of Electronic Science and Technology: research on the construction of Civic Government of Brand Management Course (No. JGB202114), and Doctoral research initiation project of Guilin University of Electronic Science and Technology: Research on the incentive mechanism of knowledge sharing in online medical communities (No. US20001Y).

## Conflict of Interest

The authors declare that the research was conducted in the absence of any commercial or financial relationships that could be construed as a potential conflict of interest.

## Publisher's Note

All claims expressed in this article are solely those of the authors and do not necessarily represent those of their affiliated organizations, or those of the publisher, the editors and the reviewers. Any product that may be evaluated in this article, or claim that may be made by its manufacturer, is not guaranteed or endorsed by the publisher.
